# A lipidomic view of SARS-CoV-2

**DOI:** 10.1042/BSR20210953

**Published:** 2021-08-05

**Authors:** Kourosh H. Ebrahimi, James S.O. McCullagh

**Affiliations:** Chemistry Research Laboratory, Department of Chemistry, University of Oxford, Oxford, U.K.

**Keywords:** COVID-19, HDL, Immune system, lipids, Metabolomics, SARS-CoV-2

## Abstract

The global pandemic of severe acute respiratory syndrome coronavirus 2 (SARS-CoV-2), which started in late 2019, has caused huge social and economic losses. A growing number of investigators are focusing on understanding the interaction of SARS-CoV-2 with host cellular processes to find therapeutic approaches. New data suggest that lipid metabolism may play a significant role in regulating the response of immune cells like macrophages to viral infection, thereby affecting the outcome of the disease. Therefore, understanding the role of lipid metabolism could help develop new therapeutic approaches to mitigate the social and economic cost of coronavirus disease 2019 (COVID-19).

Viral infections are a growing threat to human health and the economy, highlighting the need to understand their interaction with the host. The emergence of COVID-19 has accelerated this need. A growing number of investigators are working to understand how the causative agent of this disease n,amely severe acute respiratory syndrome coronavirus 2 (SARS-CoV-2) i,nteracts with the human during infection. After the virus enters the respiratory tract, the first barrier for viral entry to the host cells (epithelial cells) is the mucus, which consists of heavily glycosylated mucins and healthy bacteria. *In silico* studies predict that proteins [1] or natural products (NPs) [2] produced by these bacteria can bind to the spike glycoprotein of SARS-CoV-2, interfering with viral entry to the host cells. This interference is because the virus uses its spike glycoprotein to invade and infect host cells. Specifically, the proteolytic activity of the transmembrane protease, serine 2 (TMPRSS2) induces conformational changes in the closed state of the spike glycoprotein leading to the formation of an open state, in which the receptor-binding domain (RBD) of the spike glycoprotein is exposed [3]. Subsequently, the RBD binds to the host-cell surface receptor angiotensin-converting enzyme 2 (ACE2) for viral entry to the cells and infection [3,4]. ACE2 receptor is present in several organs, including the heart, lungs, kidneys, and the gastrointestinal tract [5]. After entry to the cell, the virus hijacks host cellular machinery to reproduce, and at the same time, the host immune system is activated to limit replication of the virus. Lipids play a central role in the life cycle of SARS-CoV-2, which is an envelope virus surrounded by a lipid bilayer, and in the immune response. In the host cells, lipid droplets (LDs) are involved in different steps of the viral replication process, for example, by acting as a platform for the replication of viral genomic material [6]. The LDs formation is induced in innate immune cells (such as macrophages) to support the host immune response [7]. The formation of LDs requires triglycerides (TGs) and phosphatidylcholine (PC). In authentic macrophages, the endogenous biosynthesis of these metabolites can be stimulated through inhibition of the glycolytic enzyme glyceraldehyde 3-phosphate dehydrogenase (GAPDH) [8,9] by the antiviral enzyme RSAD2 (also known as viperin) [10]. Additionally, free cholesterol (FC) and phospholipids (PLs) efflux of macrophages in response to the blood levels of high-density lipoprotein (HDL) and lipid-free apolipoprotein A1 (ApoA1) in the blood contributes to systemic lipid homeostasis [11,12]. However, how the regulation of cellular lipid metabolism is translated into the changes observed in systemic lipid homeostasis (blood levels of different types of lipids, HDL, very low-density lipoprotein (VLDL), and ApoA1) during viral infection is not yet understood (Figure 1).

**Figure 1:**
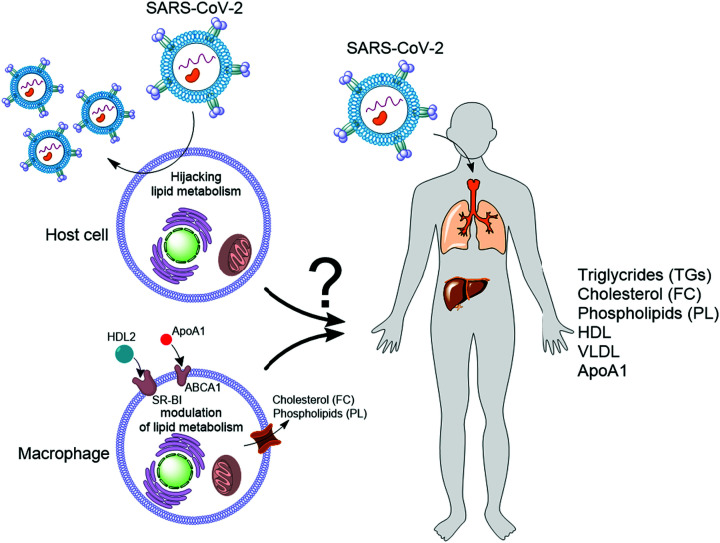
The link between cellular lipid metabolism and systemic lipid homeostasis is not clear SARS-CoV-2 infects host cells and hijacks lipid metabolism for replication. In the innate immune cells like macrophages synthesis of TGs are induced and these cells secrete TGs, cholesterol, and PLs in response to the blood levels of HDL and ApoA1. How these changes at the cellular level are translated to the systemic lipid metabolism and blood levels of TGs, FC, PLs, HDL, VLDL, and ApoA1 during SARS-CoV-2 infection is not clear.

While SARS-CoV-2 does not cause severe disease in many individuals, in those above 65 years, the excess death overwhelming increases to ∼94% [13]. Besides aging, other risk factors (Figure 2) that could determine the severity of the infection by SARS-CoV-2 include metabolic disorders such as obesity [14] and diabetes [15], and imbalances in the population of commensal microbiota [1], which are at the interface of host metabolism and immune response [16]. Therefore, a better understanding of how and why COVID-19 has such a massive impact among aged populations and those with other risk factors will help develop new therapeutic approaches and improve current treatments. Aging and other risk factors of COVID-19 are associated with metabolic changes affecting the function of the immune system. A common metabolic feature of the conditions related to these risk factors is dysfunction in lipid metabolism (Figure 2). Firstly, some metabolic products of gut microbiota like L-lactate regulate lipid metabolism in enterocytes [17]. The low bacterial richness can lead to a decrease in the levels of HDL and HDL-cholesterol (HDL-C) [18–20]. Secondly, the capacity of various tissues, particularly skeletal muscle, to oxidize fat decreases with age. This decrease may be the reason for increased production and accumulation of VLDL and some TGs [21,22]. Aging also remodels HDL. Specifically, the antioxidant activity of HDL from the elderly is reduced, and these defective HDLs are rapidly taken up by macrophages [23]. Thirdly, many studies have shown altered lipid metabolism in diabetes patients. For example, in insulin resistance individuals with Type 2 diabetes (T2D), the lipoprotein lipase (LPL) is reduced leading to a decrease in plasma HDL-C and an increase in TGs [24] and accumulation of VLDL. Finally, several studies show that the levels of HDL and HDL-C are significantly lower in individuals characterized as obese [25,26]. Thus, it appears that a common feature of the risk factors of COVID-19 is low levels of or dysfunctional HDL. The ability of HDL to dampen inflammatory signals in diabetes patients is reduced [27], and HDL can induce eicosanoid production to modulate the immune response [28]. Immune cells like macrophages show a change in lipid metabolism upon activation [29], and lipids such as eicosanoids produced by the immune cells play an essential role in regulating the immune response to pathogens [30]. These studies suggest that an imbalance in HDL production or function and lipid metabolism could contribute to the dysfunction of the immune system and the severity of COVID-19 disease. Recent studies support this proposal: (i) dysregulation of eicosanoids, lipids produced by the innate immune cells, and a shift in serum lipids are shown to be linked to SARS-CoV-2 infection [31], (ii) lipidomic studies of COVID-19 patients have revealed a positive direct correlation between lower HDL and severity and mortality rate of the disease [32], and (iii) dysregulation of lipid metabolism is correlated with pathological inflammation and severity of the COVID-19 disease [33].

**Figure 2:**
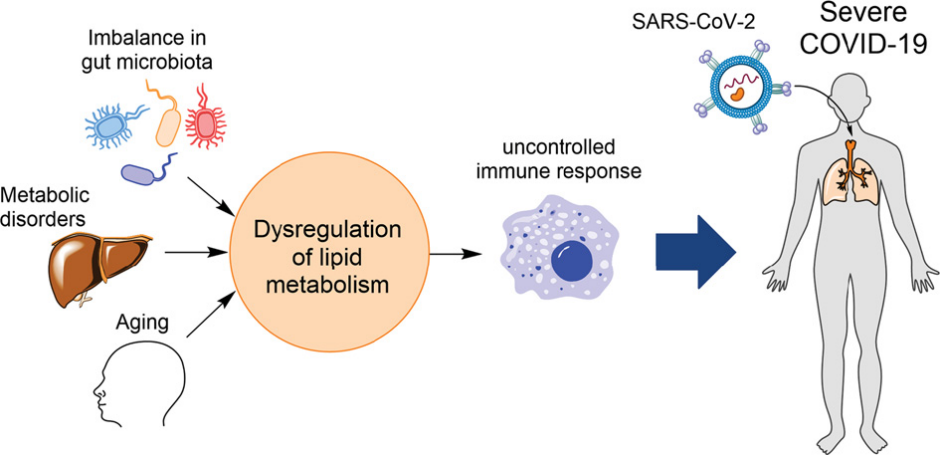
Dysregulation of lipid metabolism links the risk factors of COVID-19 disease to the dysfunction of immune cells like macrophages Different risk factors of COVID-19 such as imbalance in commensal microbiota, metabolic disorders like diabetes and obesity, and aging are characterized by dysregulation of lipid metabolism. This imbalance in lipid metabolism causes dysfunctional immune cells like macrophages, which contribute to the severity of COVID-19 disease.

Therefore, a better understanding of the interplay between metabolism, specifically cellular and systemic lipid metabolism, and SARS-CoV-2 infection is essential to develop new therapeutic approaches and mitigate the social and economic cost of the COVID-19 disease. In this respect, untargeted metabolomics using liquid chromatography-mass spectrometry (LC-MS) is a powerful approach to identify different lipids and measure their changes due to a physiological/pathophysiological condition in various matrices, including cell extracts, plasma, serum, and urine. Recently, Bai and colleagues [34] report on developing an Ultra-High-Performance Liquid Chromatography-High-Resolution Mass Spectrometry (LC–HRMS) approach for untargeted analyses of lipid metabolites in plasma samples. Using their method, they could identify and annotate ∼500 lipids in plasma. They used their approach in combination with statistical analysis to compare the difference in the levels of plasma lipids between cured patients with mild (MY group) or severe symptoms (SE group) and healthy controls. They observed that in the SE group vs. healthy controls, 62 and 23 lipids, and in the MY group vs. healthy controls, 51 and 34 lipids were up- and down-regulated, respectively. KEGG pathway analysis suggested differences in lipid metabolism between the SE or MY group compared with the healthy controls. The plasma levels of over 50 different lipids like TGs and diacylglycerols (DGs) increased in the SE and MY groups. In contrast, the levels of some other lipids like cholesteryl esters (ChEs) and lyso-phosphatidylethanolamines (LPEs) decreased. The finding that some TGs increased in the cured patients in both SE and MY groups is consistent with another recent report showing that the blood level of TGs was slightly higher in COVID-19 survivors than non-survivors [32].

Enrichment of HDL with TGs enhances HDL conversion [35], a process catalyzed by phospholipid transfer protein (PLTP) [11]. In HDL conversion, PLTP catalyzes fusion of two HDL3 forming HDL2 and releasing lipid-free ApoA1, a precursor to pre-β-HDL formation [11]. ApoA1 interacts with ATP-binding cassette A1 (ABCA1), mediating FC and PLs efflux forming pre-β-HDL [12]. HDL2 and HDL3 are two subfractions of HDL [36], and PL-rich spherical HDL2 is the ideal lipoprotein for scavenger receptor class B1 (SR-BI)-mediated cholesterol efflux [11]. Interestingly the blood level of FC in COVID-19 survivors is significantly higher than that in non-survivors [32]. Collectively, based on the available data, we propose a mechanistic model linking cellular lipid metabolism to systemic lipid homeostasis affecting the outcome of COVID-19 disease (Figure 3). Specifically, in individuals with no or minor liver damage (Figure 3A), there is a continuous efflux of TGs, FC, and PLs. These lipids are consumed for HDL biosynthesis. TG-rich HDL3 enhances the formation of HDL and HDL-mediate FC and PLs efflux, which regulates the response of macrophages [37]. Cholesterol (FC) efflux induces pro-inflammatory and anti-inflammatory responses of macrophages [37]. Damage to the liver could abolish its function in producing TGs, VLDL, and HDL (Figure 3B). As a result, consumption of TGs for regulation of HDL also decreases. Meantime, the efflux of FC and PLs continues, but their consumption for the biosynthesis of HDL reduces. Therefore, FC and PLs accumulate. This imbalance in systemic lipid homeostasis causes an uncontrolled immune response decreasing the survival rate. This model is consistent with reports showing that disruption of cholesterol biosynthesis by COVID-19 causes an imbalanced immune response and ‘cytokine storm’ leading to pulmonary damage [38]. The cause of damage to the liver can be a viral infection of this organ, which expresses the ACE2 receptor, or independent of viral infection via other factors such as diet. For example, in mice, high sucrose levels can cause hepatic steatosis [39], which is the accumulation of lipids in the liver. In the long term, the condition can lead to an advanced form of non-alcoholic fatty liver disease [40].

**Figure 3:**
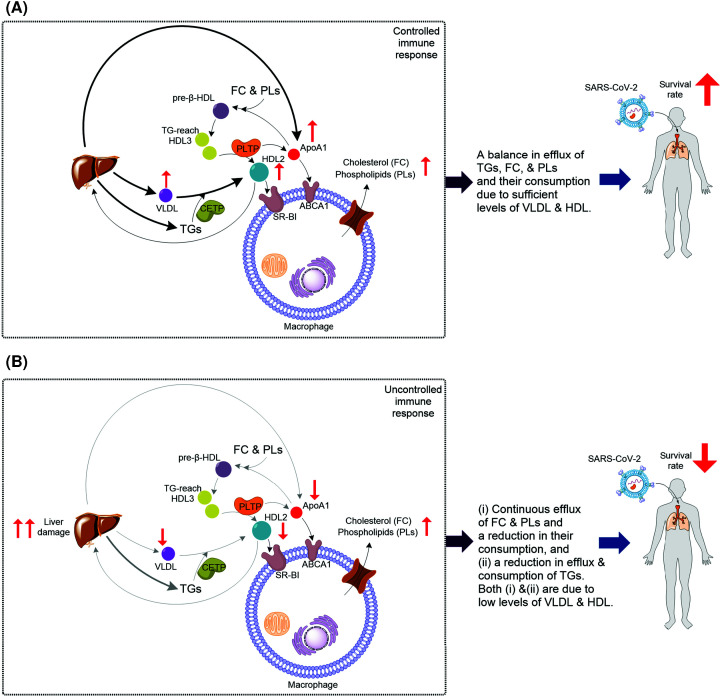
The postulated model linking cellular lipid metabolism to systemic lipid homeostasis and the outcomes of COVID-19 disease (**A**) When HDL level is sufficient, TGs, FC, and PLs produced by immune cells are consumed. Therefore, their blood levels are balanced leading to a controlled immune response and positive outcome. Enrichment of HDL3 with TGs enhances HDL conversion: fusion of two HDL3 by enzymatic activity of PLTP and formation of HDL2 with concomitant release of ApoA1. HDL2 is taken up by liver, and HDL2 and ApoA1 stimulate cholesterol efflux in macrophages. This response has both pro-inflammatory and anti-inflammatory effects. Thus, HDL regulates macrophage function to induce a controlled immune response increasing the survival rate of COVID-19 patients. (**B**) When there is not a sufficient level of HDL, which could be caused by severe liver damage or other factors, or when HDL is defective, e.g. due to aging, TGs, FC, and PE are produced by immune cells but are not consumed. This results in an uncontrolled immune response and negative outcome for patients. Both in (A,B) the change in level of TGs is the result of the net difference between efflux/production and consumption. Thus, in both (A,B) the level of TGs could increase. It is possible that the types of TGs involved in (A) are different than those involved in (B).

In summary, the study by Bai and colleagues [34] and other recent reports provide evidence in support of the model outlined in Figure 3, which links cellular lipid metabolism and systemic lipid homeostasis to the survival rates of COVID-19 patients. To further elucidates the model's validity, many fundamental questions related to lipid metabolism must be addressed. For example, which specific type of TGs or subfraction of HDL was most significantly modulated in patients who made a full recovery compared with those who did not? How does the lipid composition of HDL change during the process of infection and recovery? Which type of TGs and/or lipids are associated with macrophage function? The untargeted LC-MS approach developed by Bai and colleagues is a valuable tool to help in investigating the role of lipids in immune response and macrophage function before, during, and after treatment of COVID-19 patients. Such studies are likely to provide new insight into the importance of the specific type of lipids and lipid metabolism for a controlled and effective immune response to SARS-CoV-2 infection. We predict that the outcomes of these studies will provide a better understanding of the role of lipid metabolism in viral infection and therefore support the development of new therapeutic approaches to mitigate the ongoing social and economic cost caused by the emerging variants of SARS-CoV-2.

## Open Access

Open access for this article was enabled by the participation of University of Oxford in an all-inclusive *Read & Publish* pilot with Portland Press and the Biochemical Society under a transformative agreement with JISC.

## Abbreviations

ACE2: angiotensin-converting enzyme 2
ApoA1: apolipoprotein A1
COVID-19: coronavirus disease 2019
FC: free cholesterol
LC–MS: liquid chromatography–mass spectrometry
PL: phospholipid
PLTP: phospholipid transfer protein
RBD: receptor-binding domain
SARS-CoV-2: severe acute respiratory syndrome coronavirus 2
TG: triglyceride
VLDL: very low-density lipoprotein
